# Czech and Slovak Members of Religious Institutes: Their Health in Comparison to the General Population

**DOI:** 10.3390/ijerph18199944

**Published:** 2021-09-22

**Authors:** Dana Jaksicova, Lukas Novak, Vit Husek, Peter Tavel, Klara Malinakova

**Affiliations:** Olomouc University Social Health Institute, Palacky University in Olomouc, 771 11 Olomouc, Czech Republic; lukas.novak@oushi.upol.cz (L.N.); vit.husek@upol.cz (V.H.); peter.tavel@oushi.upol.cz (P.T.); klara.malinakova@oushi.upol.cz (K.M.)

**Keywords:** spirituality, consecrated person, health, religious institutes, Czech, Slovak

## Abstract

This study examines the general health of consecrated persons (CP) in the Czech Republic (CZ) and in Slovakia (SK) compared to control samples of the Czech population. The sample of 293 CP participants (age: *M* = 47.52, *SD* = 9.57, females: 78.88%, 180 Czechs, 213 Slovaks) was compared with two control samples, one of which was nationally representative. Comparing CP with the general population, we measured the frequency of recent health complaints, the occurrence of chronic illnesses, general health and the individual chronotype. Compared to the representative sample, CP had a higher chance of suffering from pelvis minor pain and obesity but a lower chance of diabetes. Furthermore, CP had higher odds of having worse general health. Comparing “larks” with “night owls” among CP, the “night owls” had a significantly higher chance of suffering from worse general health. “Night owl” CP also seem to suffer more from backache and depression/anxiety and to have more problems with falling asleep. Compared to the overall society, CP in CZ and SK tend to have similar or worse general health. The results differ from the findings in the US, pointing to the positive health effects of the spiritual experience and structured daily routine of CP. Thus, this study shows the importance of more detailed research on the way of life of Czech and Slovak CP to determine the factors with the most negative health effects.

## 1. Introduction

Persons who have consecrated themselves through their lifelong commitment to God and to a certain religious institute represent a minority group belonging mostly to the Roman Catholic Church [1,2]. This group differs from the majority society in several aspects, and its way of life gives rise to many questions [3,4]. The fundamental role of spirituality [5] and religiosity [6], a hierarchically structured community and subordination to authority, binding statutes and a daily routine [7,8], gender uniformity and resignation regarding sexual relationships and family life [9] are among the main specifics of living in a religious institute. A high level of social engagement [10], as well as a certain social isolation and/or self-exclusion from the mainstream [11,12], is also expected. Members of a Catholic order can be regarded as a homogenous population with a number of common characteristics on the individual and socioeconomic levels [13].

Research on health among consecrated people (CP) attracts some attention, but studies are sparse and rather ambiguous. The majority of existing studies have positive conclusions. Religious order members were reported to be at lower risk of mental health disorders [14], more successful in achieving physical and mental well-being [15] and able to systematically care for their health [16]. A prevalence of positive emotions was observed among nuns [17] as well as a higher level of personal happiness [18] and satisfaction with work engagement [19], and a lower tendency towards professional burnout [20]. Several studies chose religious communities as a sample of people living a meaningful and cognitive stimulating life and found that they had a lower risk of Alzheimer’s disease or dementia [21,22]. This corresponds to the findings about the CP’s increased ability to age meaningfully [23] and findings about their longevity [24,25]. 

In contrast, a few studies point to some deficits in the health practices of religious order members [26]. These deficits can include the danger of exhausting spiritual resources through excessive work engagement [10] or the impact of community conflicts on the health of individuals [13]. Furthermore, because the daily routine of religious communities is usually strongly oriented towards getting up early and going to bed early, without considering individual chronotype, we also suppose that persons with late circadian timing, so-called “night owls”, are, to some extent, handicapped by this regime. As opposed to “larks” with an early circadian timing, “night owls” prefer to stay up and to work long in the evening and have problems waking up and being active early in the morning [27]. “Night owls” living in a religious community with a structured daily routine may suffer from lack of sleep, which may have a negative impact on their health [28]. 

However, the existing results cannot be fully generalized because there is a need for more data from different countries and social-political contexts. To date, most studies have been carried out in the USA, which is the only country with some systematic research in CP and results regarding the general health of CP. Some of the studies quoted above come from Poland, Germany and Italy, but they were mostly oriented towards issues related to the mental health of CP and not their health in general. In all these countries, despite the advanced secularization in some of them, the presence and social engagement of religious institutes have an established tradition and a high level of public credit, which seems to play an important role [14]. On the contrary, data from secular post-communist countries are missing, as are data from African, Asian or South American communities. We presume there are also some other biases to consider, such as a high level of social desirability and non-representative samples, because it is rare for all community members to complete the voluntary questionnaire, and healthier and more active persons are more likely to participate. 

The current study examines the general health characteristics of religious brothers and sisters in the former Czechoslovakia compared to control samples of the Czech population. As the country with the highest percentage (76.4%) of religiously unaffiliated people in the world (Pew Research Center 2014), the Czech Republic (CZ) has a not very supportive milieu towards the Roman Catholic Church and religious institutes. Slovakia (SK) traditionally belongs to the Catholic countries (62% Roman Catholics and 13.4% atheists in the 2011 census), and CP are more numerous and more appreciated than they are in the Czech Republic. Nevertheless, the modern history of these two countries is firmly connected, and convents and monasteries in both countries are still carrying the consequences of the long persecution, overwork and forced isolation that occurred during the communist regime. Given these facts, we may expect, compared to previous studies, a higher level of adverse health-affecting factors and lower well-being and life satisfaction scores among members of Czech and Slovak religious communities, presuming some slight differences between the Czechs and Slovaks. 

Therefore, the aim of this study is to explore the possible associations between membership in a convent or monastery religious community in two post-communist countries and the general health characteristics of individuals. Further, we will assess if a person’s individual chronotype can play some role in the health of CP.

## 2. Methods

### 2.1. Measures

#### 2.1.1. Health Complaints

The frequency of recent health complaints was assessed using a six-item measure: headache, stomachache, backache, intestinal problems, trouble falling asleep, dizziness. The question was: *In the past month, how often have you had the following issues?* Each item was answered on a five-point scale: never (1), about once or twice (2), approximately once a week (3), more than once a week (4), every day (5). For analytical purposes, participant’s responses were dichotomized. Answers ranging from 1: *never* up to 3: *approximately once a week* were recoded as *not many times per week*, and answers ranging from 4: *more than once a week,* up to 5: *every day* were recoded as *many times a week*.

#### 2.1.2. Long Lasting Illnesses

For the occurrence of chronic illnesses, an 18-item measure was used, introduced by the question: *Do you have a long-term illness or disability? Please tick all that apply to you.* The illnesses are listed in Table 2.

#### 2.1.3. General Health

General health was assessed using a composite variable, created by summing up the number of chronic illnesses. This variable was consequently divided into several categories based on the following approach: having < 1disease was classified as “no diseases”; 1–2 diseases was classified as “few diseases”, 3–5 diseases as “several diseases” and more than 6 as “many diseases”.

#### 2.1.4. Chronotype

Daily energy was measured by choosing between two possibilities—“an early bird” (or “lark”), who wakes up early but is tired in the evening, or “a night owl”, who has problems with waking up early but enjoys working in the evening.

### 2.2. Participants

#### 2.2.1. Sample One

The first sample (*n* = 1800, Age: *M* = 46.41, *SD* = 17.4, Females: 51.28%) consisted of participants from a nationally representative Czech sample on the study of health, life experience, attitudes and lifestyle collected in 2016 [29]. In this dataset, we did not find that subjects responded incongruently to the control items, i.e., feeling the presence of God despite being non-religious or an atheist. Thus, no participant was excluded from the dataset.

#### 2.2.2. Sample Two

The second sample was collected in April 2020 as a survey made in the Czech population during the first COVID-19 lockdown. From the original dataset (*n* = 1263), we excluded 120 participants who responded incongruently to three repeatedly asked questions and those who were deemed speeders, i.e., time spent filling in the questionnaire was < 10 min. The three control questions included age (difference > 2 years), weight and height (difference > 2 kilogram and centimeters). Hence, the number of participants was 1143. Based on the results of the outliers screening procedure (see statistical analysis section), we also removed two subjects who responded to a large number of questions in the same way (*n* = 2). Therefore, the final number of participants was 1141 (Age: *M* = 49.2, *SD* = 16.73, Females: 46.45%).

#### 2.2.3. Sample Three

A sample of Catholic order members in the Czech Republic and in the Slovak Republic was recruited to take part in a survey regarding various aspects of modern consecrated life. The respondents were recruited by contacting the major superiors of all male and female religious institutes in both countries. After six weeks, information about the survey was sent directly into the local communities to increase the number of respondents. The research was conducted under the auspices of the Conference of Major Religious Superiors of the Czech and Slovak Republic. The superiors were asked to spread an online or a paper-and-pencil questionnaire among the members of their communities and to support its completion. Data were collected from March to May 2021. This sample initially consisted of 497 participants. In the first step, we excluded participants (*n* = 4) who were classified as speeders, i.e., who finished the questionnaire, which typically lasts more than 30 min, in <10 min. After this exclusion, 493 participants remained. We also removed participants who filled out the questionnaire multiple times (*n* = 63), resulting in (*n* = 430) subjects. Participants who were not Czech or Slovak were excluded (*n* = 37), resulting in 393 subjects (Age: *M* = 47.52, *SD* = 9.57, Females: 78.88%). This sample consisted of 180 Czech participants and 213 Slovak participants. No uniform response pattern was detected in this sample. The mean duration of being part of a religious community was 24.45.

### 2.3. Statistical Analysis

As suggested by the Shapiro–Wilk test and by histograms, the normality assumption was broken in all samples. Thus, non-parametric methods were used. The homogeneity of the variances was equal in all samples, as indicated by the Breusch–Pagan test. Since the null hypotheses of the MCAR test in all our surveys were not rejected, we deleted missing values listwise. Outliers were explored using the Median Absolute Deviation (MAD), and outliers identified by MAD were consequently screened. If there were signs of a uniform pattern of responding, i.e., answering a number of items in the same manner, outliers were removed from the dataset.

To explore the differences in health status among CP and non-CP, we compared long-lasting illnesses of CP to chronic illnesses of participants from the representative sample in logistic regression models. In these models, long-lasting illnesses were set as the dependent variables. The grouping variable distinguishing CP from non-CP was the regressor. Covariates consisted of gender, education and age. Ordinal logistic regression was used to compare CP and non-CP in general health. The same regression type was applied to explore associations between chronotype and general health in CP. In the ordinal regression models, the following variables were controlled for: age, education, gender and length of time spent in a religious institute. The Brant test indicated that the proportional odds assumption held for each of the ordinal regression models. A variance inflation factor (VIF) was used to assess multicollinearity in all regression models. VIF values < 10 indicated an acceptable degree of association between variables [30]. Bonferroni correction was used to correct *p*-values in all regression models. When the significance was lost after correction, we used the term “trends” to describe the relationships after the correction. The R software (Version 4.0.3, R Core Team, Vienna, Austria) [31] was utilized for all analyses.

## 3. Results

Table 1 depicts the basic sociodemographic characteristics of the study samples.

### 3.1. Chronic Illness Differences

Table 2 presents the prevalence of chronic diseases among the study samples, and Table 3 presents differences in chronic diseases between CP and the representative sample. A significant positive relationship was revealed between being a CP and a lower probability of suffering from diabetes in the crude effect. However, there was a positive relationship between being a CP and a higher chance of obesity in the crude and adjusted effect, pain in the pelvis minor in both the crude and adjusted effects and thyroid disease in the crude and adjusted effects. After Bonferroni correction, thyroid disease was non-significant.

### 3.2. Health Complaints

Table 4 refers to the associations between health complaints in CP as compared to Sample 2. Logistic regression indicated a significant relationship between CP and sleep problems: in the crude effect, CP had lower odds of having trouble falling asleep. No further significant associations were found.

### 3.3. General Health

Ordinal logistic regression revealed that CP who were “night owls” had a significantly higher chance of lower general health as compared to “early birds” in terms of crude effect (OR 1.53; 95% CI (1.02, 2.30); *p* = 0.039). In the adjusted effect, however, this result was non-significant (OR 1.45; 95% CI (0.96, 2.21); *p* = 0.078).

In the next step, we compared CP with the representative sample in terms of their general health and found that CP had significantly higher odds of having lower general health in the crude effect (OR 1.36; 95% CI (1.09, 1.69); *p* = 0.007). Moreover, in the adjusted effect, the odds of having lower general health slightly increased (OR 1.39; 95% CI (1.07, 1.81); *p* = 0.013).

### 3.4. Chronotype and Health Complaints

Table 5 shows the associations between “early birds” and “night owls” in health complaints in the CP sample. The following trends were found after Bonferroni correction: “night owls” had a higher probability of suffering from problems of falling asleep and backache as compared to “early birds” (in both crude and adjusted effect).

### 3.5. Chronotype and Chronic Illnesses

Table 6 represents the results of the logistic regression comparing “night owl CP” to “early bird CP”. Although all results were non-significant after Bonferroni correction, several trends may be observed: night owls, compared to early birds, had higher odds of developing chronic arthritis (adjusted effect) and anxiety/depression (crude and adjusted effect).

## 4. Discussion

The aim of the study was to assess the relation between living as a CP in religious institutes in the Czech Republic and in the Slovak Republic and general health. Compared to a nationally representative Czech sample, the results showed a lower probability of suffering from diabetes in CP. However, CP were found to be at a higher risk of obesity and pain in the pelvis minor (women CP) and probably also thyroid disease. Furthermore, we discovered no significant results in terms of the health complaints of CP in comparison with the control sample. Only the item “trouble falling asleep” in the religious sample was close to the significance threshold. Moreover, CP had significantly more chronic diseases. More specifically, when focusing only on CP, we found a higher risk of suffering from chronic illnesses for “night owls” compared to “larks”, though only in the crude effect. “Night owl CP” seem to suffer more from arthritis, backache and depression/anxiety and have more problems with falling asleep. 

The association between being a CP and a higher risk of obesity (Table 3) can be explained by several reasons. According to some findings, a higher tendency towards obesity is observed among believers in general [32]. Further, although there are no specialized studies in this social group, some general findings can be applied, considering the reality of religious communities. Regarding the high-performance orientation, which has been enrooted for many decades, particularly in apostolic religious congregations [33], we can assume a lack of sport and physical exercise, as well as a lack of sleep, which is often connected with unhealthy eating habits [34]. Furthermore, some religious communities still tend to follow the old, undeviating rules, which do not support individual diets [35]. In this case, all members are supposed to eat the same food, which may not be appropriate for everybody. Performance pressure is also related to a higher level of stress, which possibly also contributes to unregulated eating [36,37]. Moreover, elevated levels of the stress hormone cortisol may further influence one’s metabolism [38]. The high-performance orientation and stress can also contribute to the higher tendency of CP to suffer from thyroid disease, because chronic stress seems to be an important factor leading to this illness [39]. 

However, although a higher risk of diabetes could be expected among CP due to the previously described higher risk of obesity, which is one of the main risk factors for diabetes [40], our results did not support this presumption. These findings may be explained by the fact that another factor that significantly contributes to the development of diabetes is smoking [40], which, however, is quite rare among CP. Women CP are usually non-smokers [41] and there is only a small percentage of smokers among priest and male CP [42]. However, more data would be needed to support this potential explanation. 

Moreover, we found two or three-times higher risk of pelvis minor pain among religious sisters. There may be several reasons for this. A life in celibacy includes a higher risk of psychosomatic complaints caused by libido-suppression and a higher risk of psychosexual problems [43]. Further, we can argue that issues regarding sexuality are, to some extent, still taboo in religious institutes, because this approach is deeply enrooted in the mentality of elderly sisters, who also tend towards self-denial and ignore their health problems [33]. Therefore, it may be challenging for some consecrated women to regularly visit a gynecologist and deal with emerging health complaints in a timely manner. According to the latest findings of Nygaard et al. [44], in the case of chronic pelvic pains, early intervention is important to successfully reduce the complaints. It remains a task for future research to study the extent of traumatic experiences with sexual abuse among CP compared with a sample of non-CP, because this might be another possible reason for the chronic minor pelvis pain in CP [45]. 

Our finding of a lower general tendency of CP, as compared to the general population, to have problems falling asleep remained close to the significance threshold and more data would be needed to confirm it. However, religious community members with a “night owl” chronotype reported sleep disturbances significantly more often than “lark CP”. A common daily routine in a religious community is strictly oriented to getting up early and going to bed early, which is favorable for “larks” and inconvenient for “owls”, who have to adapt to this routine. To some extent, shifting sleep/wake timings in “night owls” to earlier hours may have a positive effect on their performance and mental health [27]. However, their biorhythm may still cause difficulties with falling asleep too early. As a consequence, some “owl brothers/owl sisters” seem to give up going to bed early and continue working late in the evening, according to their chronotype. However, they have to respect the given regime and to get up early to attend the community morning prayer. This may result in a chronic lack of sleep in this group of CP, possibly leading to other health issues.

Several studies exploring the individual chronotype concluded that the definite evening types—“night owls”—show higher rates of metabolic dysfunction and cardiovascular disease and are at a higher mortality risk [46]. Our sample is too small to discuss this topic; however, evening types were shown to have worse health characteristics. The connection between being a “night owl CP” and a higher risk of depression is consistent with general findings about this chronotype [27]. Furthermore, these CP may be stressed by the permanent lack of sleep, which can contribute to the development of depression. The higher backache tendency in “night owl CP” may be a psychosomatic consequence of a higher stress level [47]. The higher risk of arthritis in “night owl CP” does not correspond with the previous findings about rheumatoid arthritis patients, showing an earlier circadian rhythm [48]. However, our sample is not large enough and we would need more data to confirm this potential association.

When comparing Czech and Slovak CP with the representative Czech sample regarding general health, we found a significantly higher risk of having worse general health for CP. Moreover, these findings are supported by a trend, however insignificant, that could be observable in most of the chronic diseases in our study. These findings correspond to our hypothesis that, compared with the results from the USA [22,24], there are more negative health-effecting factors in CP in CZ and SK. In both cases, the comparison was carried out between CP and the general population of the same country; therefore, this cannot be seen as a possible result of different general health conditions in these two parts of the world, connected, e.g., with certain effects of colonization and the “melting pot” society on health in the USA. Rather, it seems to be connected with a different mentality and lifestyle of CP in the USA compared to CZ/SK, which may be influenced by the major society and the historical, cultural and ecclesiastical background. The delayed and only partial reception of post-conciliar changes after Vaticanum II causes a particularly higher level of conservativism in religious communities, which is typical for post-communist countries [49,50]. Czech/Slovak CP still seem to tend towards the traditional performance-oriented lifestyle characterized by strict self-denial [51]. Thus, possible reasons for the worse general health in Czech/Slovak CP could be overwork, stress and a lack of active and passive rest caused by the stereotyped regime of communities that are unadapted to their actual situation and do not provide enough space for the leisure and relaxation of individuals. Perhaps the higher incidence of psychosomatic problems should also be considered. All of these hypotheses are for future research. 

### 4.1. Strengths and Limitations 

This study has several important strengths; the most important is that it is the first study about CP living in the specific milieu of two post-communist countries. The study is based on the first blanket research among CP in these countries, which covers many dimensions of consecrated life, and allows for various factors of living in a religious community to be investigated. Moreover, as a comparison, this research uses a large, nationally representative Czech sample and another large, professionally gathered, online sample, close to national characteristics. Nevertheless, not having nationally representative data from the Slovak sample represents a certain limitation of this study. We accept that the numbers could slightly change if these were to be included, as there are slight differences between Czechs and Slovaks in terms of life expectancy and the prevalence of some chronic illnesses [52]. Nevertheless, we do not suppose that these potential differences would change the finding that CP in CZ and SK, in our sample, seem to have similar or worse general health compared to the major population. Another limitation of our study is related to the fact that the CP sample is relatively small because of the general mistrust of CP towards surveys asking personal questions. We can also expect a certain selection bias in the sample, presuming that the more active, more interested and healthier CP were more likely to fill in the questionnaire. However, in this study, this selection bias seems rather to affirm the conclusion about the lower general health of CP. 

### 4.2. Implications 

Our findings suggest that psychologists, counsellors, doctors, priests, spiritual directors and other helping professionals should be better educated about the current reality of consecrated life in CZ and SK. In their work, they should focus more deeply on internal structures, daily routine, relationships, working habits and mental hygiene in religious communities. These issues should also be directly discussed with the religious formators and superiors in charge of these communities. 

Future research should be oriented towards revealing positive and negative health-effecting factors among CP, comparing CP both from local communities of one country and from different countries and cultural contexts, so that generally spread and culturally conditioned factors can be distinguished.

## 5. Conclusions

According to our findings, persons living a consecrated life in the post-communist Czech Republic and Slovak Republic tend to have similar or slightly worse general health compared to the majority society. The results differ from US findings pointing out the positive health effects of spiritual experience and structured daily routine. Thus, this study shows the importance of more detailed research on the way of life of (not only) Czech and Slovak religious communities to determine factors that could contribute to their negative health outcomes. 

## Figures and Tables

**Table 1:** Sociodemographic table.

	Sample 1	Sample 2	Sample 3 (CZ, SK)
Characteristic	N = 1800	N = 1141	N = 393
Gender			
Female	923 (51%)	530 (50%)	310 (79%)
Male	877 (49%)	523 (50%)	83 (21%)
Family status			
Not in relationship	439 (24%)	267 (25%)	
Married	929 (52%)	461 (44%)	
Divorced	158 (8.8%)	201 (19%)	
Widow/Widower	133 (7.4%)	73 (6.9%)	
In relationship	141 (7.8%)	51 (4.8%)	
Education			
Basic school	141 (7.8%)	90 (8.7%)	1 (0.3%)
Vocational school or non-maturity high school	442 (25%)	400 (39%)	12 (3.1%)
High school	854 (47%)	377 (36%)	48 (12%)
Higher vocational school or University	363 (20%)	169 (16%)	332 (84%)
Economic status			
Without work	261 (14%)	149 (14%)	
Pensioner	430 (24%)	325 (31%)	
Working	1109 (62%)	559 (54%)	
Faith			
Yes, I am a member of church	170 (9.4%)		
Yes, but I am not a member of a church	361 (20%)		
No	1004 (56%)		
No, I am convinced atheist	265 (15%)		

**Table 2:** General health and chronic illnesses among the study samples.

Characteristic	Sample 1, N = 1800	Sample 2, N = 1141	Sample 3, N = 393
ICHS	68 (3.8%)	47 (4.8%)	10 (2.9%)
Hypertension	371 (21%)	243 (25%)	69 (20%)
Stroke	20 (1.1%)	20 (2.0%)	3 (0.9%)
Asthma	166 (9.2%)	94 (9.6%)	32 (9.4%)
Cancer	36 (2.0%)	28 (2.9%)	9 (2.7%)
Diabetes	182 (10%)	117 (12%)	12 (3.5%)
Obesity	183 (10%)	218 (22%)	56 (17%)
Arthritis	121 (6.7%)	102 (10%)	29 (8.6%)
Back pain	631 (35%)	348 (35%)	131 (39%)
Gastric or duodenal ulcers	56 (3.1%)	31 (3.2%)	12 (3.5%)
Chronic lung disease	24 (1.3%)	36 (3.7%)	7 (2.1%)
Skin diseases eczema	156 (8.7%)	102 (10%)	38 (11%)
Allergy	364 (20%)	178 (18%)	83 (24%)
Migraine	223 (12%)	94 (9.6%)	42 (12%)
Pain of unclear origin	99 (5.5%)	65 (6.6%)	14 (4.1%)
Pain in the pelvis minor	68 (3.8%)	35 (3.6%)	37 (11%)
Depression/Anxiety	125 (6.9%)	102 (10%)	40 (12%)
Thyroid disease	152 (8.4%)	110 (11%)	46 (14%)
General health	1.69 (1.54)	2.01 (1.93)	1.98 (1.71)

*Note:* ICHS = Ischemic heart disease; in the general health variable, values refers to M(SD).

**Table 3:** Associations (in odds rations) between living in clerical life and chronic diseases (Sample 1 and 3).

	Pain in the Pelvis Minor	Obesity	Diabetes	Arthritis	Thyroid Disease
Crude effect	3.12 *** (2.04, 4.72)	1.75 *** (1.25, 2.41)	0.33 *** (0.17, 0.57)	1.30 (0.84, 1.95)	1.70 ** (1.19, 2.40)
Adjusted effect	1.99 * (1.16, 3.40)	1.85 ** (1.23, 2.78)	0.46 * (0.23, 0.86)	1.62 (0.94, 2.74)	1.57 * (1.01, 2.45)
					
	Depression/Anxiety	Migraine	Pain of unclear origin	Cancer	
Crude effect	1.79 ** (1.22, 2.59)	1.00 (0.70, 1.41)	0.74 (0.40, 1.27)	1.34 (0.60, 2.68)	
Adjusted effect	1.58 (0.98, 2.53)	0.74 (0.49, 1.12)	0.77 (0.38, 1.46)	1.69 (0.64, 4.21)	
					
	Hypertension	Ischemic heart disease	Stroke	Back pain	
Crude effect	0.98 (0.73, 1.31)	0.77 (0.37, 1.45)	0.79 (0.19, 2.33)	1.17 (0.92, 1.48)	
Adjusted effect	1.26 (0.89, 1.79)	1.72 (0.72, 3.85)	1.91 (0.36, 8.45)	1.27 (0.95, 1.70)	
					
	Gastric or duodenal ulcers	Chronic lung disease	Skin diseases eczema	Allergy	
Crude effect	1.14 (0.58, 2.08)	1.56 (0.62, 3.47)	1.33 (0.90, 1.92)	1.28 (0.97, 1.67)	
Adjusted effect	1.28 (0.57, 2.74)	1.51 (0.50, 4.29)	1.31 (0.82, 2.07)	1.15 (0.83, 1.60)	

*Note: p* < 0.05 *, *p* < 0.01 **, *p* < 0.001 ***, The adjusted effect was calculated using the following variables as covariates: Age, Gender and Education. Values in brackets indicate the 95% confidence interval. After Bonferroni correction, the first three results in the first row remained significant. Other relationships were non-significant.

**Table 4:** Associations (in odds rations) between being a CP and health complaints in the last month (Samples 2 and 3).

	Trouble Falling Asleep	Headache	Stomachache
Crude effect	0.65 * (0.45, 0.92)	0.87 (0.54, 1.36)	0.68 (0.33, 1.28)
Adjusted effect	0.84 (0.52, 1.34)	0.99 (0.53, 1.83)	0.61 (0.25, 1.43)
			
	Backache	Intestinal problems	Dizziness
Crude effect	0.99 (0.74, 1.31)	1.31 (0.78, 2.16)	0.85 (0.41, 1.64)
Adjusted effect	1.00 (0.67, 1.48)	1.37 (0.67, 2.81)	1.41 (0.53, 3.60)

*Note: p* < 0.05 *, The adjusted effect was calculated using the following variables as covariates: Age, Gender and Education. Values in brackets indicate the 95% confidence interval.

**Table 5:** Associations (in odds rations) between “early birds” and “night owls” and health complaints (Sample 3).

	Headache	Stomachache	Backache
Crude effect	0.76 (0.32, 1.70)	0.70 (0.18, 2.37)	1.73 * (1.05, 2.86)
Adjusted effect	0.72 (0.31, 1.36)	0.70 (0.18, 2.42)	1.74 * (1.04, 2.92)
			
	Intestinal problems	Trouble falling asleep	Dizziness
Crude effect	1.67 (0.71, 4.02)	2.55 ** (1.34, 4.99)	1.51 (0.45, 5.33)
Adjusted effect	1.60 (0.68, 3.91)	2.59 ** (1.35, 5.11)	1.44 (0.42, 5.12)

*Note: p* < 0.05 *, *p* < 0.01 **, The adjusted effect was calculated using the following variables as covariates: Age, Gender, Education and years spent in a religious institute. Values in brackets indicates the 95% confidence interval. After Bonferroni correction all results were non-significant.

**Table 6:** Associations (in odds rations) between “early bird CP” and “night owl CP” and chronic diseases (Sample 3).

	Gastric or Duodenal Ulcers	Chronic Lung Disease	Skin Diseases Eczema	Allergy	Migraine
Crude effect	2.23 (0.66, 8.64)	1.67 (0.36, 8.60)	1.27 (0.65, 2.52)	0.93 (0.56, 1.54)	1.15 (0.60, 2.20)
Adjusted effect	2.12 (0.63, 8.27)	1.66 (0.36, 8.55)	1.28 (0.64, 2.55)	0.94 (0.57, 1.57)	1.04 (0.53, 2.01)
					
	Depression/Anxiety	Ischemic heart disease	Obesity	Back pain	
Crude effect	2.02 * (1.04, 4.03)	0.82 (0.21, 2.93)	1.41 (0.79, 2.52)	1.02 (0.66, 1.59)	
Adjusted effect	2.00 * (1.03, 4.01)	0.54 (0.11, 2.30)	1.47 (0.81, 2.67)	1.01 (0.64, 1.60)	
					
	Hypertension	Diabetes	Arthritis	Asthma	
Crude effect	1.26 (0.74, 2.15)	0.88 (0.26, 2.82)	2.55 * (1.17, 5.89)	1.93 (0.93, 4.14)	
Adjusted effect	1.25 (0.71, 2.18)	0.86 (0.25, 2.78)	3.49 ** (1.43, 9.31)	1.89 (0.90, 4.08)	
					
	Pain of unclear origin	Pain in the pelvis minor	Cancer	Thyroid disease	
Crude effect	0.32 (0.07, 1.06)	0.64 (0.31, 1.29)	0.35 (0.05, 1.46)	1.16 (0.62, 2.17)	
Adjusted effect	0.36 (0.08, 1.21)	0.63 (0.29, 1.30)	0.29 (0.04, 1.34)	1.03 (0.54, 1.95)	

*Note: p* < 0.05 *, *p* < 0.01 **, The adjusted effect was calculated using the following variables as covariates: Age, Gender, Education and number of years spent in a religious institute. Values in brackets indicate the 95% confidence interval. After Bonferroni correction, all results were non-significant. The variable *stroke* was excluded from the analysis because the regression model containing this variable did not converge.

## Data Availability

The data and code used in this study are openly available in [Open Science Framework] at [https://doi.org/10.17605/OSF.IO/TV6M5] (accessed on 20 September 2021), reference number [tv6m5].
